# 7-Chloro-11a-phenyl-2,3,5,10,11,11a-hexa­hydro-1*H*-pyrrolo[2,1-*c*][1,4]benzodiazepine-5,11-dione

**DOI:** 10.1107/S160053680800408X

**Published:** 2008-02-13

**Authors:** Rafael Tamazyan, Armen Ayvazyan, Ashot Martirosyan, Gohar Harutyunyan, Vahan Martirosyan

**Affiliations:** a Molecular Structure Research Center, National Academy of Sciences RA, Azatutyan Avenue 26, 375014 Yerevan, Republic of Armenia; b Institute of Fine Organic Chemistry, National Academy of Sciences RA, Azatutyan Avenue 26, 375014 Yerevan, Republic of Armenia

## Abstract

The title compound, C_18_H_15_ClN_2_O_2_, is a potential human immunodeficiency virus type-1 (HIV-1) non-nucleoside reverse transcriptase inhibitor. The pyrrolidine ring adopts an envelope and the diazepine ring a boat conformation. In the crystal structure, two isomers (*R* and *S*) form centrosymmetric dimers *via* N—H⋯O hydrogen bonds.

## Related literature

For details of the pharmacological properties of this family of compounds, see: De Clercq (1996 ▶). For the crystal structures of some analogues of the title compound, see: Karapetyan *et al.* (2002 ▶); Tamazyan *et al.* (2002 ▶, 2007 ▶). For reference structural data, see Allen *et al.* (1987 ▶).
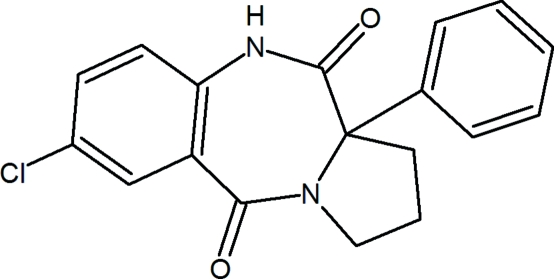


## Experimental

### 

#### Crystal data

C_18_H_15_ClN_2_O_2_
*M* *_r_* = 326.77
Triclinic, 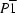

*a* = 8.9749 (18) Å
*b* = 9.2184 (18) Å
*c* = 9.912 (2) Å
α = 86.90 (3)°
β = 71.35 (3)°
γ = 88.27 (3)°
*V* = 775.8 (3) Å^3^
*Z* = 2
Mo *K*α radiation
μ = 0.26 mm^−1^
*T* = 293 (2) K
0.35 × 0.32 × 0.28 mm

#### Data collection

Enraf–Nonius CAD-4 diffractometer
Absorption correction: none
7347 measured reflections
4514 independent reflections
3148 reflections with *I* > 2σ(*I*)
*R*_int_ = 0.035
3 standard reflections frequency: 180 min intensity decay: none

#### Refinement

*R*[*F* ^2^ > 2σ(*F* ^2^)] = 0.046
*wR*(*F* ^2^) = 0.121
*S* = 1.02
4514 reflections
268 parameters
All H-atom parameters refined
Δρ_max_ = 0.27 e Å^−3^
Δρ_min_ = −0.31 e Å^−3^

### 

Data collection: *DATCOL* in *CAD-4* (Enraf–Nonius, 1988 ▶); cell refinement: *LS* in *CAD-4*; data reduction: *HELENA* (Spek, 1997 ▶); program(s) used to solve structure: *SHELXS97* (Sheldrick, 2008 ▶); program(s) used to refine structure: *SHELXL97* (Sheldrick, 2008 ▶); molecular graphics: *SHELXTL* (Sheldrick, 2008 ▶) and *ORTEPII* (Johnson, 1976 ▶); software used to prepare material for publication: *SHELXTL*.

## Supplementary Material

Crystal structure: contains datablocks global, I. DOI: 10.1107/S160053680800408X/sj2462sup1.cif

Structure factors: contains datablocks I. DOI: 10.1107/S160053680800408X/sj2462Isup2.hkl

Additional supplementary materials:  crystallographic information; 3D view; checkCIF report

## Figures and Tables

**Table 1:** Hydrogen-bond geometry (Å, °)

*D*—H⋯*A*	*D*—H	H⋯*A*	*D*⋯*A*	*D*—H⋯*A*
N4—H4⋯O12^i^	0.85 (2)	2.35 (2)	2.997 (2)	133 (2)

Symmetry code: (i) 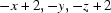.

**Fig. 1.**
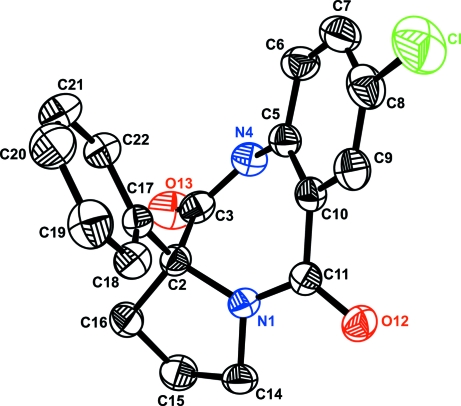
A view of molecule with the atomic numbering scheme. Displacement ellipsoids are drawn at the 50% probability level. H atoms are omitted for clarity.

**Fig. 2.**
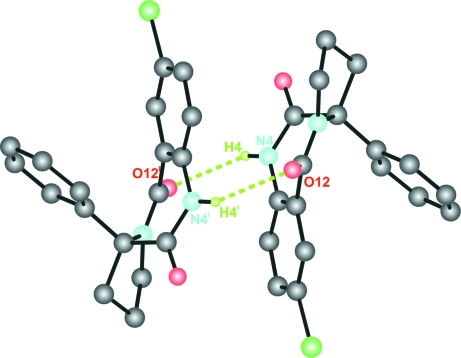
The formation dimers of molecules via hydrogen bonding. For clarity only those H atoms participating in hydrogen bonding are depicted. Symmetry code: (i) 2 - x, -y, 2 - z

## supplementary crystallographic information

### Comment

Interest in X-ray structural investigation of title compound, (I), Fig. 1, is stimulated by its potentially HIV-1 RT inhibition properties. These compounds belong to a family of non-nucleoside reverse transcriptase inhibitors (NNRTIs).

All intramolecular interatomic distances in molecule are in good agreement with their mean statistical values (Allen *et al.*, 1987). In the crystal structure dimers are formed by (*R* and S) optical isomers of molecules of (I) *via* O12···H4^i^—N4^i^ and O12^i^···H4—N4 double hydrogen bonding (Table 1, Fig.2).

### Experimental

A solution of 2-phenyl-2-pyrrolidinecarboxylic acid (0.01 mol) and 6-chloro-1,4-dihydro-2*H*-3,1-benzoxazine-2,4-dione (0.01 mol) in dry DMF (5 ml) was boiled for 4 h. After cooling in an ice bath the title compound formed as a colourless precipitate and was separated by filtration and washed with ethylacetate. The compound as synthesized was a racemic mixture. Crystals were grown from an ethanol solution of the compound.

### Refinement

Hydrogen atoms were located in a difference map and refined freely with isotropic thermal parameters.

### Crystal data

**Table:**

C_18_H_15_ClN_2_O_2_	*Z* = 2
*M**_r_* = 326.77	*F*(000) = 340
Triclinic, *P*1	*D*_x_ = 1.399 Mg m^−^^3^
Hall symbol: -P 1	Mo *K*α radiation, λ = 0.71073 Å
*a* = 8.9749 (18) Å	Cell parameters from 22 reflections
*b* = 9.2184 (18) Å	θ = 12.6–16.6°
*c* = 9.912 (2) Å	µ = 0.26 mm^−^^1^
α = 86.90 (3)°	*T* = 293 K
β = 71.35 (3)°	Prism, colourless
γ = 88.27 (3)°	0.35 × 0.32 × 0.28 mm
*V* = 775.8 (3) Å^3^	

### Data collection

**Table:**

Enraf–Nonius CAD-4 diffractometer	*R*_int_ = 0.035
Radiation source: fine-focus sealed tube	θ_max_ = 30.0°, θ_min_ = 2.2°
graphite	*h* = −12→12
θ/2θ scans	*k* = −12→12
7347 measured reflections	*l* = −13→13
4514 independent reflections	3 standard reflections every 180 min
3148 reflections with *I* > 2σ(*I*)	intensity decay: none

### Refinement

**Table:**

Refinement on *F*^2^	Primary atom site location: structure-invariant direct methods
Least-squares matrix: full	Secondary atom site location: difference Fourier map
*R*[*F*^2^ > 2σ(*F*^2^)] = 0.046	Hydrogen site location: difference Fourier map
*wR*(*F*^2^) = 0.121	All H-atom parameters refined
*S* = 1.02	*w* = 1/[σ^2^(*F*_o_^2^) + (0.0496*P*)^2^ + 0.2193*P*] where *P* = (*F*_o_^2^ + 2*F*_c_^2^)/3
4514 reflections	(Δ/σ)_max_ < 0.001
268 parameters	Δρ_max_ = 0.27 e Å^−^^3^
0 restraints	Δρ_min_ = −0.31 e Å^−^^3^

### Special details

**Table:**

Geometry. All e.s.d.'s (except the e.s.d. in the dihedral angle between two l.s. planes) are estimated using the full covariance matrix. The cell e.s.d.'s are taken into account individually in the estimation of e.s.d.'s in distances, angles and torsion angles; correlations between e.s.d.'s in cell parameters are only used when they are defined by crystal symmetry. An approximate (isotropic) treatment of cell e.s.d.'s is used for estimating e.s.d.'s involving l.s. planes.
Refinement. Refinement of *F*^2^ against ALL reflections. The weighted *R*-factor *wR* and goodness of fit *S* are based on *F*^2^, conventional *R*-factors *R* are based on *F*, with *F* set to zero for negative *F*^2^. The threshold expression of *F*^2^ > σ(*F*^2^) is used only for calculating *R*-factors(gt) *etc*. and is not relevant to the choice of reflections for refinement. *R*-factors based on *F*^2^ are statistically about twice as large as those based on *F*, and *R*- factors based on ALL data will be even larger.

### Fractional atomic coordinates and isotropic or equivalent isotropic displacement parameters (Å^2^)

**Table:**

	*x*	*y*	*z*	*U*_iso_*/*U*_eq_	
C2	0.70626 (17)	0.27403 (17)	1.00932 (16)	0.0333 (3)	
C3	0.66409 (19)	0.11255 (18)	1.04089 (17)	0.0389 (3)	
C5	0.91077 (18)	0.04305 (16)	0.85230 (16)	0.0343 (3)	
C6	0.9468 (2)	−0.05252 (19)	0.74145 (19)	0.0432 (4)	
C7	1.0802 (2)	−0.0350 (2)	0.6255 (2)	0.0488 (4)	
C8	1.1788 (2)	0.0798 (2)	0.61835 (19)	0.0470 (4)	
C9	1.1492 (2)	0.1717 (2)	0.72885 (19)	0.0418 (4)	
C10	1.01455 (17)	0.15430 (16)	0.84775 (16)	0.0332 (3)	
C11	0.99809 (17)	0.25083 (15)	0.96767 (16)	0.0319 (3)	
C14	0.8259 (2)	0.37447 (19)	1.17444 (18)	0.0391 (3)	
C15	0.6494 (2)	0.3646 (2)	1.24567 (19)	0.0493 (4)	
C16	0.5879 (2)	0.3677 (2)	1.11921 (19)	0.0448 (4)	
C17	0.71935 (17)	0.32632 (16)	0.85722 (16)	0.0335 (3)	
C18	0.8016 (2)	0.45256 (19)	0.79868 (18)	0.0405 (4)	
C19	0.8104 (2)	0.5045 (2)	0.6632 (2)	0.0519 (4)	
C20	0.7365 (3)	0.4332 (2)	0.5842 (2)	0.0571 (5)	
C21	0.6523 (3)	0.3101 (2)	0.6421 (2)	0.0562 (5)	
C22	0.6436 (2)	0.2556 (2)	0.7781 (2)	0.0439 (4)	
Cl	1.33908 (7)	0.10803 (8)	0.46554 (6)	0.0735 (2)	
H4	0.745 (2)	−0.072 (2)	0.987 (2)	0.052 (6)*	
H6	0.874 (2)	−0.135 (2)	0.747 (2)	0.047 (5)*	
H7	1.101 (3)	−0.097 (2)	0.551 (2)	0.062 (6)*	
H9	1.219 (2)	0.246 (2)	0.725 (2)	0.049 (5)*	
H18	0.851 (2)	0.504 (2)	0.853 (2)	0.048 (5)*	
H19	0.864 (3)	0.590 (3)	0.625 (2)	0.065 (7)*	
H20	0.745 (3)	0.481 (2)	0.482 (3)	0.064 (6)*	
H21	0.599 (3)	0.259 (2)	0.595 (2)	0.058 (6)*	
H22	0.584 (2)	0.174 (2)	0.816 (2)	0.051 (6)*	
H14A	0.856 (2)	0.473 (2)	1.1500 (19)	0.040 (5)*	
H15A	0.608 (3)	0.445 (3)	1.311 (2)	0.065 (6)*	
H16A	0.596 (2)	0.467 (2)	1.080 (2)	0.052 (6)*	
H14B	0.886 (2)	0.328 (2)	1.233 (2)	0.048 (5)*	
H15B	0.623 (2)	0.270 (2)	1.301 (2)	0.054 (6)*	
H16B	0.484 (3)	0.331 (2)	1.139 (2)	0.055 (6)*	
N1	0.85316 (15)	0.29356 (14)	1.04452 (13)	0.0327 (3)	
N4	0.77286 (16)	0.01605 (16)	0.96731 (15)	0.0390 (3)	
O12	1.11592 (13)	0.28878 (12)	0.99545 (13)	0.0410 (3)	
O13	0.54222 (16)	0.07208 (16)	1.12946 (15)	0.0604 (4)	

### Atomic displacement parameters (Å^2^)

**Table:**

	*U*^11^	*U*^22^	*U*^33^	*U*^12^	*U*^13^	*U*^23^
C2	0.0302 (7)	0.0378 (8)	0.0323 (7)	−0.0007 (6)	−0.0102 (5)	−0.0035 (6)
C3	0.0373 (8)	0.0440 (9)	0.0352 (8)	−0.0102 (6)	−0.0106 (6)	−0.0001 (6)
C5	0.0389 (8)	0.0298 (7)	0.0351 (7)	0.0016 (6)	−0.0134 (6)	0.0008 (6)
C6	0.0543 (10)	0.0354 (8)	0.0433 (9)	0.0004 (7)	−0.0199 (8)	−0.0040 (7)
C7	0.0610 (11)	0.0468 (10)	0.0399 (9)	0.0125 (8)	−0.0178 (8)	−0.0093 (8)
C8	0.0423 (9)	0.0553 (11)	0.0373 (8)	0.0095 (8)	−0.0055 (7)	0.0000 (8)
C9	0.0352 (8)	0.0438 (9)	0.0437 (9)	0.0006 (7)	−0.0092 (7)	0.0006 (7)
C10	0.0324 (7)	0.0316 (7)	0.0359 (7)	0.0017 (5)	−0.0118 (6)	−0.0001 (6)
C11	0.0342 (7)	0.0263 (7)	0.0361 (7)	−0.0030 (5)	−0.0131 (6)	0.0040 (5)
C14	0.0480 (9)	0.0360 (8)	0.0367 (8)	0.0009 (7)	−0.0174 (7)	−0.0070 (7)
C15	0.0494 (10)	0.0602 (12)	0.0365 (9)	0.0050 (9)	−0.0102 (7)	−0.0112 (8)
C16	0.0394 (9)	0.0540 (11)	0.0407 (9)	0.0087 (8)	−0.0115 (7)	−0.0118 (8)
C17	0.0310 (7)	0.0363 (8)	0.0343 (7)	0.0047 (6)	−0.0119 (6)	−0.0050 (6)
C18	0.0417 (9)	0.0405 (9)	0.0418 (9)	−0.0001 (7)	−0.0168 (7)	−0.0032 (7)
C19	0.0563 (11)	0.0507 (11)	0.0463 (10)	−0.0007 (9)	−0.0147 (8)	0.0080 (8)
C20	0.0711 (13)	0.0641 (13)	0.0382 (9)	0.0087 (10)	−0.0213 (9)	−0.0014 (9)
C21	0.0687 (13)	0.0618 (12)	0.0508 (11)	0.0060 (10)	−0.0354 (10)	−0.0148 (9)
C22	0.0462 (9)	0.0440 (9)	0.0467 (9)	−0.0005 (7)	−0.0215 (8)	−0.0068 (8)
Cl	0.0554 (3)	0.0986 (5)	0.0498 (3)	0.0051 (3)	0.0065 (2)	−0.0053 (3)
N1	0.0349 (6)	0.0337 (6)	0.0318 (6)	−0.0002 (5)	−0.0134 (5)	−0.0037 (5)
N4	0.0418 (7)	0.0314 (7)	0.0417 (7)	−0.0078 (5)	−0.0106 (6)	0.0026 (6)
O12	0.0371 (6)	0.0362 (6)	0.0532 (7)	−0.0047 (4)	−0.0189 (5)	−0.0019 (5)
O13	0.0519 (8)	0.0631 (9)	0.0522 (8)	−0.0217 (7)	0.0050 (6)	−0.0049 (6)

### Geometric parameters (Å, °)

**Table:**

C2—N1	1.4858 (19)	C14—C15	1.517 (3)
C2—C17	1.527 (2)	C14—H14A	0.956 (19)
C2—C3	1.538 (2)	C14—H14B	0.99 (2)
C2—C16	1.539 (2)	C15—C16	1.522 (3)
C3—O13	1.217 (2)	C15—H15A	0.99 (2)
C3—N4	1.356 (2)	C15—H15B	1.00 (2)
C5—C10	1.395 (2)	C16—H16A	0.97 (2)
C5—C6	1.396 (2)	C16—H16B	0.96 (2)
C5—N4	1.407 (2)	C17—C22	1.387 (2)
C6—C7	1.375 (3)	C17—C18	1.393 (2)
C6—H6	1.010 (19)	C18—C19	1.380 (3)
C7—C8	1.385 (3)	C18—H18	0.95 (2)
C7—H7	0.93 (2)	C19—C20	1.378 (3)
C8—C9	1.374 (3)	C19—H19	0.94 (2)
C8—Cl	1.7370 (19)	C20—C21	1.376 (3)
C9—C10	1.398 (2)	C20—H20	1.06 (2)
C9—H9	0.93 (2)	C21—C22	1.392 (3)
C10—C11	1.490 (2)	C21—H21	0.92 (2)
C11—O12	1.2391 (18)	C22—H22	0.92 (2)
C11—N1	1.340 (2)	N4—H4	0.85 (2)
C14—N1	1.472 (2)		
			
N1—C2—C17	113.17 (12)	C14—C15—C16	102.64 (15)
N1—C2—C3	106.65 (12)	C14—C15—H15A	111.5 (13)
C17—C2—C3	113.09 (13)	C16—C15—H15A	113.8 (13)
N1—C2—C16	101.84 (12)	C14—C15—H15B	110.0 (12)
C17—C2—C16	111.26 (13)	C16—C15—H15B	109.7 (12)
C3—C2—C16	110.18 (14)	H15A—C15—H15B	109.1 (18)
O13—C3—N4	121.24 (16)	C15—C16—C2	104.63 (14)
O13—C3—C2	122.47 (16)	C15—C16—H16A	107.3 (12)
N4—C3—C2	116.26 (13)	C2—C16—H16A	107.6 (12)
C10—C5—C6	119.46 (15)	C15—C16—H16B	115.7 (13)
C10—C5—N4	123.50 (14)	C2—C16—H16B	109.7 (13)
C6—C5—N4	116.96 (15)	H16A—C16—H16B	111.4 (18)
C7—C6—C5	120.84 (17)	C22—C17—C18	118.84 (15)
C7—C6—H6	119.9 (11)	C22—C17—C2	121.50 (15)
C5—C6—H6	119.3 (11)	C18—C17—C2	119.56 (14)
C6—C7—C8	119.40 (17)	C19—C18—C17	120.52 (17)
C6—C7—H7	120.2 (14)	C19—C18—H18	119.7 (12)
C8—C7—H7	120.4 (14)	C17—C18—H18	119.8 (12)
C9—C8—C7	120.77 (17)	C20—C19—C18	120.54 (19)
C9—C8—Cl	120.40 (15)	C20—C19—H19	119.1 (14)
C7—C8—Cl	118.82 (15)	C18—C19—H19	120.3 (14)
C8—C9—C10	120.23 (17)	C21—C20—C19	119.41 (18)
C8—C9—H9	120.2 (12)	C21—C20—H20	124.0 (12)
C10—C9—H9	119.6 (12)	C19—C20—H20	116.5 (12)
C5—C10—C9	119.18 (15)	C20—C21—C22	120.71 (19)
C5—C10—C11	123.97 (14)	C20—C21—H21	123.0 (13)
C9—C10—C11	116.69 (14)	C22—C21—H21	116.3 (14)
O12—C11—N1	121.43 (14)	C17—C22—C21	119.96 (18)
O12—C11—C10	120.38 (14)	C17—C22—H22	120.9 (13)
N1—C11—C10	118.18 (13)	C21—C22—H22	119.1 (13)
N1—C14—C15	103.15 (14)	C11—N1—C14	121.27 (13)
N1—C14—H14A	110.2 (11)	C11—N1—C2	126.30 (12)
C15—C14—H14A	110.6 (11)	C14—N1—C2	112.36 (12)
N1—C14—H14B	109.6 (11)	C3—N4—C5	128.71 (14)
C15—C14—H14B	112.6 (11)	C3—N4—H4	113.7 (14)
H14A—C14—H14B	110.4 (15)	C5—N4—H4	116.9 (14)
			
N1—C2—C3—O13	118.43 (17)	C3—C2—C17—C22	23.3 (2)
C17—C2—C3—O13	−116.54 (18)	C16—C2—C17—C22	−101.29 (18)
C16—C2—C3—O13	8.7 (2)	N1—C2—C17—C18	−38.96 (19)
N1—C2—C3—N4	−59.63 (17)	C3—C2—C17—C18	−160.38 (14)
C17—C2—C3—N4	65.40 (18)	C16—C2—C17—C18	75.00 (18)
C16—C2—C3—N4	−169.39 (14)	C22—C17—C18—C19	−1.4 (2)
C10—C5—C6—C7	−2.5 (2)	C2—C17—C18—C19	−177.78 (16)
N4—C5—C6—C7	−179.40 (15)	C17—C18—C19—C20	0.7 (3)
C5—C6—C7—C8	−0.6 (3)	C18—C19—C20—C21	0.6 (3)
C6—C7—C8—C9	3.3 (3)	C19—C20—C21—C22	−1.2 (3)
C6—C7—C8—Cl	−175.46 (14)	C18—C17—C22—C21	0.8 (3)
C7—C8—C9—C10	−2.9 (3)	C2—C17—C22—C21	177.10 (16)
Cl—C8—C9—C10	175.88 (13)	C20—C21—C22—C17	0.5 (3)
C6—C5—C10—C9	2.9 (2)	O12—C11—N1—C14	−6.6 (2)
N4—C5—C10—C9	179.60 (14)	C10—C11—N1—C14	172.75 (13)
C6—C5—C10—C11	−172.22 (14)	O12—C11—N1—C2	170.27 (13)
N4—C5—C10—C11	4.5 (2)	C10—C11—N1—C2	−10.4 (2)
C8—C9—C10—C5	−0.3 (2)	C15—C14—N1—C11	−165.56 (14)
C8—C9—C10—C11	175.20 (15)	C15—C14—N1—C2	17.18 (18)
C5—C10—C11—O12	141.38 (15)	C17—C2—N1—C11	−50.6 (2)
C9—C10—C11—O12	−33.9 (2)	C3—C2—N1—C11	74.38 (18)
C5—C10—C11—N1	−38.0 (2)	C16—C2—N1—C11	−170.11 (15)
C9—C10—C11—N1	146.80 (14)	C17—C2—N1—C14	126.50 (14)
N1—C14—C15—C16	−34.22 (19)	C3—C2—N1—C14	−108.52 (14)
C14—C15—C16—C2	39.5 (2)	C16—C2—N1—C14	6.98 (17)
N1—C2—C16—C15	−28.50 (18)	O13—C3—N4—C5	172.76 (16)
C17—C2—C16—C15	−149.37 (15)	C2—C3—N4—C5	−9.1 (2)
C3—C2—C16—C15	84.38 (18)	C10—C5—N4—C3	43.9 (2)
N1—C2—C17—C22	144.75 (15)	C6—C5—N4—C3	−139.36 (17)

### Hydrogen-bond geometry (Å, °)

**Table:**

*D*—H···*A*	*D*—H	H···*A*	*D*···*A*	*D*—H···*A*
N4—H4···O12^i^	0.85 (2)	2.35 (2)	2.997 (2)	133 (2)

Symmetry codes: (i) −*x*+2, −*y*, −*z*+2.
